# Brain Networks Modulation during Simple and Complex Gait: A “Mobile Brain/Body Imaging” Study

**DOI:** 10.3390/s24092875

**Published:** 2024-04-30

**Authors:** Gaia Bonassi, Mingqi Zhao, Jessica Samogin, Dante Mantini, Roberta Marchese, Luciano Contrino, Paola Tognetti, Martina Putzolu, Alessandro Botta, Elisa Pelosin, Laura Avanzino

**Affiliations:** 1 Department of Neuroscience, Rehabilitation, Ophthalmology, Genetics, Maternal and Child Health, University of Genoa, 16132 Genoa, Italy; gaia.bonassi@unige.it; 2 Research Center for Motor Control and Neuroplasticity, KU Leuven, 3001 Leuven, Belgium; zhaomq@lzu.edu.cn (M.Z.); jessica.samogin@kuleuven.be (J.S.); dante.mantini@kuleuven.be (D.M.); 3 Gansu Provincial Key Laboratory of Wearable Computing, School of Information Science and Engineering, Lanzhou University, Lanzhou 730000, China; 4 IRCCS Ospedale Policlinico San Martino, 16132 Genoa, Italy; roberta.marchese@hsanmartino.it (R.M.); botta.alessandro90@gmail.com (A.B.); lavanzino76@gmail.com (L.A.); 5 S.C. Medicina Fisica e Riabilitazione Ospedaliera, Azienda Sanitaria Locale Chiavarese, 16043 Chiavari, Italy; lcontrino@asl4.liguria.it (L.C.); paolatognetti.pt@gmail.com (P.T.); 6 Department of Experimental Medicine, Section of Human Physiology, University of Genoa, Viale Benedetto XV 3, 16132 Genoa, Italy; martina.putzolu@unige.it

**Keywords:** high-density electroencephalography, gait, dual task, cognitive network, affective network

## Abstract

Walking encompasses a complex interplay of neuromuscular coordination and cognitive processes. Disruptions in gait can impact personal independence and quality of life, especially among the elderly and neurodegenerative patients. While traditional biomechanical analyses and neuroimaging techniques have contributed to understanding gait control, they often lack the temporal resolution needed for rapid neural dynamics. This study employs a mobile brain/body imaging (MoBI) platform with high-density electroencephalography (hd-EEG) to explore event-related desynchronization and synchronization (ERD/ERS) during overground walking. Simultaneous to hdEEG, we recorded gait spatiotemporal parameters. Participants were asked to walk under usual walking and dual-task walking conditions. For data analysis, we extracted ERD/ERS in α, β, and γ bands from 17 selected regions of interest encompassing not only the sensorimotor cerebral network but also the cognitive and affective networks. A correlation analysis was performed between gait parameters and ERD/ERS intensities in different networks in the different phases of gait. Results showed that ERD/ERS modulations across gait phases in the α and β bands extended beyond the sensorimotor network, over the cognitive and limbic networks, and were more prominent in all networks during dual tasks with respect to usual walking. Correlation analyses showed that a stronger α ERS in the initial double-support phases correlates with shorter step length, emphasizing the role of attention in motor control. Additionally, β ERD/ERS in affective and cognitive networks during dual-task walking correlated with dual-task gait performance, suggesting compensatory mechanisms in complex tasks. This study advances our understanding of neural dynamics during overground walking, emphasizing the multidimensional nature of gait control involving cognitive and affective networks.

## 1. Introduction

Walking, a fundamental human motor activity, encompasses a complex interplay of neuromuscular coordination and cognitive processes. Gait requires a complex orchestration of interconnected neural systems that manage balance, rhythmic walking, and adaptation to the task and environment [1]. Gait disturbances can significantly impact personal independence and quality of life, potentially leading to mobility limitations and increased risk of falls, particularly among the elderly population [2,3] and neurodegenerative patients [4]. Since gait is a significant indicator of overall health and functional well-being, understanding the neural underpinnings of gait control has garnered substantial interest.

Traditionally, research studies have primarily used biomechanical analyses to examine kinematic and kinetic patterns during gait [5]. Recent advances in neuroimaging techniques, including positron emission tomography, functional magnetic resonance imaging, and single photon emission computer tomography, have provided new insights into the cortical and subcortical regions involved in gait control [6,7,8,9]. However, these methods often lack the temporal resolution required to capture the rapid neural dynamics associated with gait.

High-density electroencephalography (hdEEG) provides a unique avenue to explore the real-time neural processes underlying gait, allowing researchers to investigate brain activity with high temporal precision. Specifically, wireless EEG systems can be used in combination with kinematic and/or electromyography sensors placed over the limbs for mobile brain/body imaging (MoBI) experiments [10,11]. Moreover, by combining hdEEG with novel, realistic head modeling methods [12] it is possible to analyze neural oscillations in the source space, similarly to brain imaging tools [13].

Our group has recently validated an approach with MoBI that combines hdEEG with kinematic measurements, with an accurate and efficient myogenic artifact removal [11,14]. We focused our analysis, at the source level, on sensorimotor and premotor areas, and the cerebellum. We observed event-related desynchronization and synchronization (ERD/ERS) modulation across gait phases in the α (8–13 Hz), β (13–30 Hz), and γ (30–50 Hz) bands, especially in the primary motor cortex [11] during treadmill walking. These findings demonstrated that hdEEG recordings, in combination with appropriate artifact suppression and source-level analysis, allow for the investigation of ERD/ERS dynamics across the gait cycle. Low-frequency bands, such as the theta band, were not explored in our previous study since evidence in the literature reported ERD/ERS modulation in the α, β, and γ bands during robot-assisted gait training [15], treadmill walking [16], and pedaling [17]. Some limitations emerged from our previous study. First, we focused on a treadmill walking task, which is a controlled environment but is not as ecological as overground walking. Second, we addressed neural modulation during usual walking, analyzing specific regions of the sensorimotor cerebral network that are involved in gait control (cerebellum, primary motor cortex, thalamus, supplementary motor area). It would be of interest to study neural modulation in different circumstances (i.e., during the execution of a dual task) and to expand the EEG analysis to other cerebral networks. Indeed, it is well known that the cortical and subcortical areas involved in cognitive and affective functions are also involved in gait control [18].

Hence, the present study expands on our prior research [11] by investigating gait-related neural modulations in young, healthy individuals during two overground walking conditions: (i) usual walking and (ii) dual-task walking involving a cognitive oddball task. Neural activity and gait parameters were simultaneously recorded using a MoBI platform based on hdEEG and stereophotogrammetric motion capture, respectively. Then, ERD/ERS in α, β, and γ frequency bands from selected ROIs, including sensorimotor, cognitive, and affective networks, were extracted, and correlation analyses to explore the relationship between gait parameters and ERD/ERS intensities were also computed.

## 2. Materials and Methods

### 2.1. Experiment and Data

Seventeen healthy participants (9 females, age [mean ± SD] 28 ± 3.7 years) without any brain-related injury/disease or serious medical condition were recruited for the experiment. The experimental procedures were approved by the Ethics Committee of the Liguria Region, Italy (reference: 238/2019) and were conducted in accordance with the 1964 Helsinki Declaration and its later amendments. Prior to participation, written informed consent was provided by each participant.

### 2.2. Mobile Brain–Body Imaging Platform

For this protocol, we customized a MoBI platform, like [19], to collect hdEEG data while recording gait spatiotemporal parameters during overground walking.

The details of the MoBI platform are fully reported by Zhao and collaborators [19]. Briefly, the equipment consisted of the following:

(i) A backpack weighing approximately 1.7 kg containing an ActiCHamp EEG amplifier (Brain Products GmbH, Germany) equipped with 128 channels, a Surface Go tablet (Microsoft Corporation, Redmond, WA, United States), and a lightweight battery. The amplifier, powered by the battery, was interfaced with the tablet for reliable data storage. The EEG sensors, integrated into the cap following a standard 10/20 montage, were connected to the subject’s scalp.
(ii) The stereophotogrammetric system was connected to an EEG amplifier. A pulse signal was sent from the motion capture system to one of the auxiliary channels of the hdEEG amplifier, for offline synchronization of the two systems.

### 2.3. Experimental Paradigm

**Figure 1:**
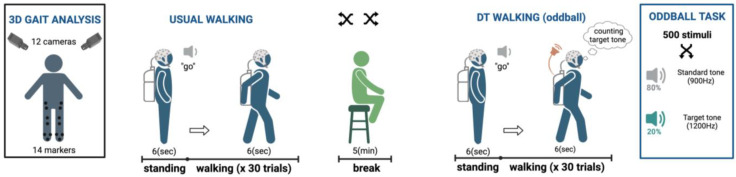
Experimental paradigm. The task design included two conditions: usual overground walking, and a dual-task condition with an oddball task during gait. The two gait tasks were randomized. Each condition consisted of 30 subsequent walking trials, in which participants stood still for 6 s (baseline), and then, after a ‘go’ sound, walked for 6 seconds, about 5 m, on a wide linear ground.

The experimental paradigm is depicted in Figure 1. The gait task comprised two conditions: usual walking, and a dual-task incorporating an oddball task during gait. Each condition consisted of 30 subsequent consecutive walking trials during which participants remained stationary for 6 seconds (baseline), before initiating walking upon hearing a ‘go’ sound, for 6 seconds, on a wide linear ground. In both conditions, subjects walked at their self-selected speed [11,19,20,21], wearing the MoBI equipment described above. During the dual-task condition (oddball task), a series of auditory stimuli (500) was played by the tablet in the backpack. A standard tone (900 Hz) and a target tone (1200 Hz) were randomly presented. Each one lasted 62 ms, with fade-in and fade-out periods of 10 ms and a loudness of about 65 dB at the participant’s ears. The inter-stimulus interval was set to randomly vary between 1000 and 1375 ms [22]. Participants were instructed to silently count the occurrences of the target tones (20% of the total stimuli) while disregarding the other tone. The oddball task, indeed, is an easy, extensively used protocol providing additional cognitive load without requiring additional physical movements or verbal responses.

### 2.4. Data Collection

We collected 128-channel hdEEG data using an ActiCHamp amplifier (Brain Products GmbH, Gilching, Germany). The hdEEG sensors, integrated into an EEG cap with standard 10/20 montage, were connected to the participant’s scalp through a conductive gel. The hdEEG data were sampled at 1 kHz frequency, using the FCz electrode as a physical reference. Electrode impedance was kept below 5 kΩ. Electrooculographic (EOG) signals were recorded to monitor for vertical (VEOG) and horizontal (HEOG) eye movement. The EOG recordings were subsequently used for EEG artifact removal.

Simultaneously with the hdEEG signals, we also collected gait spatiotemporal parameters. Three-dimensional (3D) gait analysis was performed with a 12-camera 3D motion capture system (VICON NEXUS v2.7, Oxford Metrics Group, Oxford, UK, 100 Hz). Fourteen reflective markers were positioned at specific bony landmarks of the participant according to the Human Body lower limb model (including the foot, ankle, knee, leg, and pelvis) applying the lower body Plug-in Gait model integrated into the Vicon system. The temporal jitter between the hdEEG data and acceleration signals was experimentally quantified and found to be less than 5 ms.

### 2.5. Gait Analysis

Data analysis was conducted using the Nexus 2.12 software (Vicon^®^). First, we checked data quality regarding the visibility of the markers of each participant. Gaps in marker detection (>30 samples) were identified and excluded from the analysis; gaps < 30 samples were interpolated using cubic spline fitting. Then, manual detection of gait events was performed for each cycle, including left heel strike (LHS), right heel strike (RHS), left toe off (LTO), and right toe off (RTO), following established criteria [15,20,21]. Heel strike was determined as the lowest vertical point of the heel marker during gait, and toe-off was identified as the point of maximum knee extension [23,24]. Based on these events, the gait cycle was divided into four phases [11]: initial double support (IDS) between LHS and RTO, right leg swing (RLS) between RTO and RHS, final double support (FDS) between RHS and LTO, and left leg swing (LLS) between LTO and LHS [14,25,26]. Spatiotemporal parameters, including gait speed, step length, and step width, were extracted for both the usual and dual-task conditions. Additionally, to evaluate changes in the gait parameters induced by the dual task with respect to the single task, a delta value was calculated as follows: (gait_parameter_ dual task − gait_parameter_ single task)/gait_parameter_ single task) × 100 [27].

### 2.6. EEG Analysis

The analysis involved four main steps: data pre-processing, head model creation, source localization, and ERD/ERS imaging [28,29,30].

### 2.7. Data Pre-Processing, Head Model Creation, and Source Localization

Data pre-processing, head model creation, and source localization were performed following the same pipeline as described in our previous study on this topic [11]. Data preprocessing included bad-channel correction, filtering in the frequency band [1–80 Hz], and attenuation of ocular, movement, and myogenic artifacts [14]. The head model was built using a template magnetic resonance (MR) image in combination with template electrode positions using the MR-TIM [12] and the Simbio [31]. We fed this forward model template, together with the preprocessed individual hdEEG data, into the exact low-resolution brain electromagnetic tomography (eLORETA) method [32] to obtain a three-dimensional neural signal for each voxel in the grey matter. The neural signal of each voxel was then projected to a single dimension for further analysis using principal component analysis [33].

### 2.8. ERD/ERS Imaging

Frequency-dependent modulations of neural oscillations were assessed by conducting an ERD/ERS analysis of the gait cycle on the brain signals reconstructed from selected regions of interest (ROIs). These ROIs are part of specific networks of interest in gait neurophysiology (sensorimotor, cognitive, and affective networks). The choice of the ROI was based on previous works on the sensorimotor, cognitive, and limbic networks involved in gait control [34]. We selected 17 regions of interest (ROIs) [11,35,36] included in the AAL brain atlas [37], defined in the Montreal Neurological Institute (MNI) space, each corresponding to cortical areas involved in at least one of the defined networks. The list of ROIs corresponding to each mask is reported in Table 1.

For each ROI, the MNI coordinates were first transformed into individual spaces. A representative ROI signal was acquired by calculating the first principal component of signals from the voxels within a 6 mm sphere of the individual coordinates. Then, the spectrogram of the signal was epoched according to gait cycles, warped according to gait events, averaged with events aligned to their grand mean, and corrected for baseline to generate a time-frequency map [15,20,25]. Finally, the time-frequency map was averaged in each gait phase separately for the α (8–13 Hz), β (13–30 Hz), and γ (30–50 Hz) bands to evaluate the corresponding ERD/ERS intensity.

### 2.9. Statistical Analysis

Spatiotemporal gait parameters were compared using paired *t*-tests between usual and dual-task walking conditions. The significance of the ERD/ERS intensities was evaluated using paired *t*-tests and corrected for multiple comparisons using the false discovery rate (FDR) method.

Pearson’s correlation coefficients (r) were used to identify a possible relationship between gait parameters and ERD/ERS intensities in different networks (sensorimotor, cognitive, limbic) in the different phases of gait (IDS, RLS, FDS, LLS) at the individual level. The significance level of the *t*-test was set to *p* < 0.05 and to pFDR < 0.05 after correction for multiple comparisons. All analyses were conducted with MATLAB (R2018a, Math-Works, Natick, MA, USA).

## 3. Results

### 3.1. Gait Behavioural Data

Gait parameters are reported in Table 2. For each participant, we included about 120 gait cycles for each condition. Average velocity ranged from 3.1 to 5.1 km/h for usual gait and from 2.7 to 4.9 km/h in the dual-task condition. Statistical analysis revealed a significant increase in step width during the dual-task gait compared to the usual gait (*p* always >0.05).

### 3.2. Gait-Related Neural Modulations in Sensorimotor, Cognitive, and Limbic Networks

**Figure 2:**
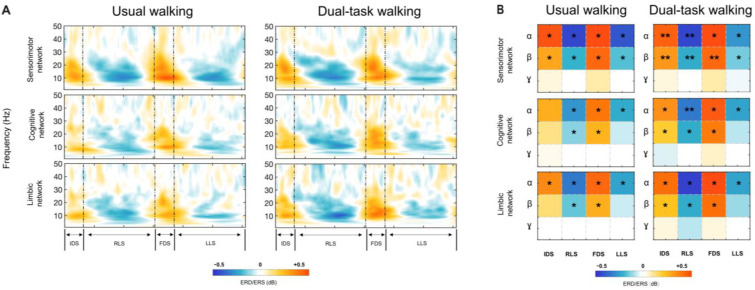
(**A**). Gait-related time-frequency analysis for the network of interest. The plot shows the average frequency-dependent modulations of neural signals in the gait cycle across participants, calculated from the neural signal of the different ROIs selected for each network. Event-related desynchronization/synchronization (ERD/ERS) during usual gait conditions and dual-task conditions are reported. The timing of gait events is indicated using dashed vertical lines. IDS, initial double support; RLS, right leg swing; FDS, final double support; LLS, left leg swing. (**B**). Significance of ERD/ERS across frequencies and gait phases of the three brain networks involved (sensorimotor, cognitive, and limbic). The values represented in the matrices are the grand mean of the ERD/ERS values, for each frequency band and gait phase. α, alpha; β, beta; γ, gamma; * pFDR < 0.05; ** pFDR < 0.01.

We extracted the ERD/ERS values of three selected brain networks (i.e., sensorimotor, SMN cognitive, CN, and limbic, LN) as a grand mean of ERD/ERS values of specific regions of interest (ROIs), selected for each network (Figure 2A). Furthermore, we tested their significance for each frequency band and gait phase, separately for the usual and dual-task gaits (Figure 2B).

Confirming our previous findings on gait-related neural oscillations in sensorimotor areas [11], we observed, in the sensorimotor network, for the α and β bands, ERS during the double-support phases (IDS and FDS) and ERD at comparable frequencies during the swing phases (LLS and RLS) (Figure 2A). Notably, this finding was also observed for the dual-task gait, with more prominent ERS in the β band in IDS and FDS (*p* = 0.006) and ERD in RLS (*p* = 0.006) in the dual-task gait, with respect to usual walking (IDS, *p* = 0.048; FDS, *p* = 0.038; RLS, *p* = 0.037) (Figure 2B).

Interestingly, for the α and β bands, ERS during the double-support phases (IDS and FDS), and ERD at comparable frequencies during the swing phases (LLS and RLS), were also observed in the cognitive and limbic networks, with different significances for usual and dual-task walking (Figure 2A,B).

In the cognitive network, more prominent ERS was observed in the dual-task gait in IDS and FDS (IDS α, *p* = 0.014; FDS α, *p* = 0.016; IDS β, *p* = 0.026; FDS β, *p* = 0.014) with respect to usual walking (IDS α, *p* = 0.08; FDS α, *p* = 0.025; IDS β, *p* = 0.13; FDS β, *p* = 0.025) (Figure 2B). Furthermore, more prominent ERD was observed in the dual-task gait in RLS (α, *p* = 0.004; β, *p* = 0.014) with respect to usual walking (α, *p* = 0.043; β, *p* = 0.025) (Figure 2B).

Finally, in the limbic network, more prominent ERS was observed in the dual-task gait in β in IDS (*p* = 0.028) with respect to usual walking (*p* = 0.21) (Figure 2B). Similar ERD for the α and β bands was observed in the limbic network during RLS and LLS in the dual-task and usual walking (Figure 2B).

The results of the ERS/ERD analysis across frequencies and gait phases showed no significant effect within the γ band across all three networks investigated.

### 3.3. Correlations between Gait Related ERD/ERS and Gait Parameters

**Figure 3:**
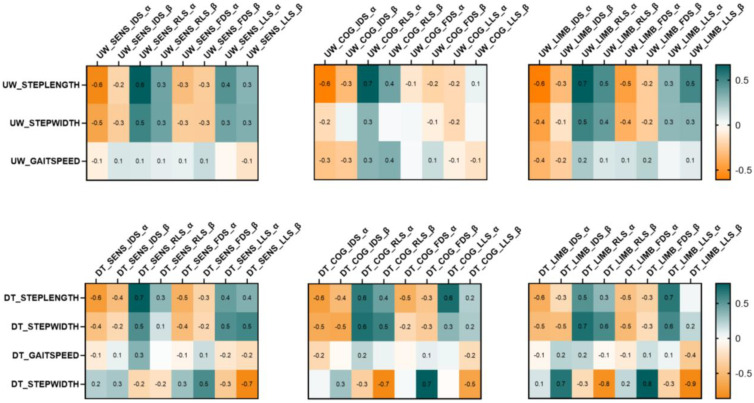
Heatmap correlation matrices reporting Pearson correlation. The pictures show the correlation matrices of ERD/ERS modulation in alpha and in beta in different events of the gait cycle collected during usual walking (**above panel**) and dual-task walking (**below panel**) and gait parameters. The color bars on the right side of the maps represent Pearson’s correlation coefficients (R). UW, usual walking; DT, dual-task walking; SENS, sensorimotor network; LIMB, limbic network; IDS, initial double support; RLS, right leg swing; FDS, final double support; LLS, left leg swing; A, alpha EEG rhythm; B, beta EEG rhythm.

Correlation results are reported in Figure 3.

In usual walking, ERS in α during IDS in the sensorimotor, limbic, and cognitive networks was negatively correlated with step length, indicating that shorter step length was associated with stronger ERS. Furthermore, in usual walking, ERD in α during RLS in sensorimotor and cognitive networks was always positively correlated with step length, indicating that a shorter step length was associated with a stronger ERD.

A similar finding was obtained in the dual-task, with ERS in α during IDS in sensorimotor, limbic, and cognitive networks that negatively correlated with step length during dual-task walking, and ERD in α during RLS in sensorimotor, limbic, and cognitive networks that positively correlated with step length during dual-task walking.

We also found significant correlations between β ERD/ERS during dual-task walking and dual-task cost of step width. Particularly, β ERS in the limbic network during IDS and FDS positively correlated with the dual-task cost of step width, whereas β ERD in the limbic network during RLS and LLS negatively correlated with the dual-task cost of step width. In the cognitive network, β ERS during FDS positively correlated with the dual-task cost of step width, whereas β ERD during RLS negatively correlated with the dual-task cost of step width. These findings indicate that in the limbic and cognitive networks stronger β ERS in double-support phases and stronger β ERD in leg-swing phases were associated with a greater increase in step width in the dual-task gait with respect to the usual gait.

## 4. Discussion

In this study, we investigated gait-related neural modulation using hdEEG during usual walking and dual-task walking during overground walking. In relation to the spatiotemporal parameters of gait, we found a significant deterioration of gait performance in the dual-task condition with respect to usual walking, which was particularly evidenced by increased step width. Consistent with our previous results [11], we found ERD/ERS modulations for the α and β bands in the sensorimotor network during overground usual walking (a difference from our previous study in which treadmill walking was considered) supporting the applicability of our MoBI approach in recording ERD/ERS modulation during overground walking.

Interestingly, we also found that the α and β band ERD/ERS modulations extended beyond the sensorimotor network, over the cognitive and limbic networks, and were more prominent in all networks during the dual-task with respect to usual walking. Finally, significant correlations were identified between the α and β band ERD/ERS modulations across networks and gait parameters.

In difference with our previous study, we found no significant γ band ERD/ERS modulations across all three networks investigated and all phases of the gait cycle. This might be due to our study focusing on the cortical network-level response to walking in both simple and dual-task conditions, whereas our previous study [11] reported gamma-band activity within specific regions of interest (ROIs), particularly the cerebellum and the thalamus.

### 4.1. ERD/ERS Modulation across Gait Cycle in Sensorimotor, Cognitive, and Limbic Networks during Usual and Dual-Task Overground Walking

The leg-swing phases were associated with ERD in the α and β bands in the sensorimotor, cognitive, and affective networks. Such an ERD during movement has been largely described in the sensorimotor cortex, and it has been suggested to be associated with an increased recruitment of neurons in specific cortical regions controlling the leg swing [38,39]. Finding such an ERD also in cognitive and affective neural structures indicates increased recruitment of neurons in areas beyond the sensorimotor cortex. A large network of cortical, basal ganglia, thalamic, cerebellar, brainstem, and spinal circuits participates in the mechanisms underlying gait (for a review see [8]). Notably, the networks subserving gait control have enlarged in recent years, encompassing, in addition to sensorimotor areas, cognitive and affective neural circuits [8]. Behaviorally, it has been shown that both emotional and cognitive states can influence gait [40,41]. For example, depression and sadness can be linked to reduced walking speed, arm swing, and vertical head motions [42]. “Sad walking” in healthy people was associated with increased neck and trunk flexion, a decreased range of motion for the limbs, and a decreased walking speed [43]. On the other hand, “joyful walking” was linked to higher trunk extension, a larger range of motion, and an increase in gait velocity [43]. The involvement of cognitive circuits in gait control is largely documented by the fact that, in pathological conditions characterized by decreased cognitive performance, gait performance is modified [44]. In addition to being common in Alzheimer’s disease (AD) patients, changes in gait can also indicate a higher likelihood of dementia in those with mild cognitive impairment (MCI) [45,46]. People with AD and MCI have been found to have several gait markers, such as shortened stride length, slow walking speed, and high variability [47,48,49]. The relationship between gait impairment and cognitive impairment may be partially explained by shared brain networks [50]. Postural control during gait may also be impacted by spatial navigation and visuospatial abilities, which are possibly compromised in people with AD and MCI [51,52].

Here, we also observed that, unlike in the leg-swing phases, the double-support phases were characterized by ERS in the α and β bands in sensorimotor, cognitive, and affective networks. Post-movement synchronization (PMS), particularly in the β band, has consistently been described on movement cessation. It is believed that PMS represents the maintenance of current motor states and consistent motor output [53,54]. Moreover, the processing of movement-related sensory afference has been linked to PMS. The latter assumption is supported by the observations that a similar phenomenon also occurs after passive movements [55,56], and that those errors related to the completed movement [57] modulate the PMS. Furthermore, PMS is associated with motor adaptation, with a PMS amplitude that simultaneously reflects cortical sensory processing and signals either the need for maintenance or the adaptation of the motor output [58]. Thus, we hypothesize that the ERS observed during the double-support phases in the sensorimotor, cognitive, and affective networks may serve for sensory processing and the maintenance or adaptation of the motor output (leg-swing phases). Neuroimaging data support the involvement of cerebral networks subserving sensory processing and planning in controlling the double-support phases. In a group of patients with gait disorders (including increased duration of the double-support phases) and age-related white matter changes [59], longer double-support phases (that is, more severe gait disturbances) are associated with reduced gait-induced perfusion changes in networks subserving sensory processing and planning, such as the supplementary motor area, visual cortex, and thalamus [59].

Additionally, we found that ERD/ERS modulations were also observed in all networks during the dual-task gait, with more prominent activity, particularly in the double-support and swing phases, for the α and β bands compared to usual walking. Dual-task walking, which combines a cognitive task with a gait task, is a daily activity that simultaneously challenges both dynamic balance and executive function. Increased task complexity and demand for attention may contribute to heightened ERD activity [60,61,62,63]. Notably, more prominent activity was observed not only in the cognitive and affective networks (given the larger cognitive demands and stressful situations associated with dual-task walking with respect to usual walking) but also in the sensorimotor network, suggesting an increased resource utilization in the sensorimotor areas. A stronger engagement in the sensorimotor cortex in the dual-task gait, with respect to usual walking, has been shown in both young people and the elderly, with cortical activity recorded with functional near-infrared spectroscopy that increased with a dual task in the premotor cortex, supplementary motor area, and primary motor area [64].

### 4.2. Correlation between ERD/ERS Oscillation and Gait Parameters

Our results showed that ERD/ERS in the α band was correlated with step parameters. Specifically, the stronger the ERS activity in the different networks during the initial double-support phase and the ERD activity in the leg-swing phases, the shorter the step length (both during the usual and dual-task walking). Alpha oscillations have been linked to attention during sensorimotor tasks in previous EEG research [65,66,67]. The α band ERD is associated with anticipatory attention [68,69] and information processing [70]. The α band ERS is associated with the control of the execution of a response, particularly over sites that exert a top-down control process [71].

This finding suggests that the α oscillations may be related not only to sensorimotor processing, but also to other functions critical for efficient motor control and, following our correlation analysis, instrumental for step-length adaptation.

In relation to the dual-task cost, we observed that, in the affective and cognitive networks, stronger β band ERS in double-support phases and β band ERD in leg-swing phases during the dual-task gait were associated with a greater dual-task cost for step width (an increase in step width in the dual-task gait with respect to usual gait). Dual-task cost, defined as the relative change from a performance executed in a single task with respect to a dual-task condition [72], is a way to measure to what extent performance diminishes in a dual-task with respect to a single task. Deterioration of gait in a dual-task performance with respect to usual gait is expected following the theories about dual-task, i.e., (i) the “bottleneck theory”, asserting that if two actions need to use common brain networks they are processed sequentially, with a consequent compromission of one task when the other one is performed [73,74], or (ii) the capacity sharing model, stating that when we perform two tasks simultaneously, the presence of competing attentional requests can result in a decline in the performance of one or both tasks due to the limited capacity of attention [75]. Correlation between step width dual-task cost and the β band ERD/ERS in affective and cognitive networks suggests a greater engagement of these networks during dual-task walking when gait performance is hindered by performing the gait task and an attentional task simultaneously. This may reflect compensatory mechanisms to face control of step width in a more complex task, requiring a larger amount of cognitive and affective control, such as in the case of the dual-task gait with respect to usual walking. A similar pattern of compensatory activity has been found in pathological conditions. As an example, in Parkinson’s disease, a larger activation of cognitive circuits has already been demonstrated during usual walking, by using functional near-infrared spectroscopy fNIRS, likely reflecting the need for the utilization of cognitive resources even in a relatively “simple” task to compensate for gait alterations [76].

## 5. Conclusions

By showing that ERD/ERS modulation in the α and β bands across the gait cycle extends beyond the sensorimotor network, over the cognitive and limbic networks, and that it is more prominent in all networks during a dual-task gait with respect to usual walking, this study advances our understanding of neural dynamics during overground walking, emphasizing the multidimensional nature of gait control. The use of MoBI with hdEEG provides valuable insights, paving the way for future research in gait-related neurophysiology. However, when considering the applicability of our findings to real-life settings, it is essential to acknowledge the potential limitations associated with the devices used in our study, including issues of comfort, ease of use, and ecological validity. While our study employed a mobile brain/body imaging platform with high-density electroencephalography, the comfort and practicality of wearing EEG caps and additional equipment during walking tasks may be a concern, particularly for certain populations (i.e., the elderly or persons with motor impairments). Furthermore, the controlled laboratory environment in which our study was conducted may limit the ecological validity of our findings in real-world scenarios. Future developments in wearable EEG technology aimed at enhancing comfort, portability, and usability could address these limitations and facilitate the translation of our monitoring protocols into more ecologically valid settings.

## Figures and Tables

**Table 1:** Montreal Neurological Institute (MNI) coordinates for selected regions of interest (ROIs).

Selected ROIs	MNI Coordinates
**Sensorimotor network**	
Putamen	R (28, 5, 2) L (−24, 4, 2)
Supplementary Motor Area (SMA)	(0, −8, 56)
Primary Motor Cortex (PMC)	R (−34, −12, 68) L (34, −12, 68)
Dorsal Premotor Cortex (DPMC)	R (34, −4, 62) L (−28, −22, 64)
Ventral Premotor Cortex (VPMC)	R (32, −8, 52) L (−26, −8, 54)
**Cognitive network**	
Dorsal Prefrontal Cortex (DLPFC)	R (45.14, 38.44, 24.49) L (−39.41, 52.62, 9.74)
Caudate Nucleus	R (15, 12, 9) L (−11, 11, 9)
**Limbic Network**	
Medial Prefrontal Cortex (MPFC)	R (18, 36, 32) L (−14, 46, 26)
Nucleus Accumbens	R (11, 11, 1) L (−8, 12, 1)

**Table 2:** Gait spatiotemporal parameters in the two experimental conditions (usual walking and dual-task walking) across participants.

	Usual Gait		Dual Task Gait		
	MEAN	SD	MEAN	SD	*p*
**Gait speed (m/s)**	1.03	0.15	1.01	0.15	0.64
**Step length (m)**	0.63	0.06	0.61	0.05	0.08
**Step width (m)**	0.18	0.04	0.19	0.03	*0.001*
**Gait speed dual task cost**	-	-	−0.96	11.8	-
**Step length dual task cost**	-	-	−3.25	5.85	-
**Step width dual task cost**	-	-	7.41	6.99	-

## Data Availability

Data are available upon request.
